# Phages from Genus *Bruynoghevirus* and Phage Therapy: Pseudomonas Phage Delta Case

**DOI:** 10.3390/v13101965

**Published:** 2021-09-30

**Authors:** Petar Knezevic, Aleksandra Petrovic Fabijan, Damir Gavric, Jovana Pejic, Zsolt Doffkay, Gábor Rakhely

**Affiliations:** 1 Department of Biology and Ecology, Faculty of Sciences, University of Novi Sad, Trg Dositeja Obradovica 3, 21 000 Novi Sad, Serbia; aleksandra.petrovic@dbe.uns.ac.rs (A.P.F.); damir.gavric@dbe.uns.ac.rs (D.G.); jovana.pejic@dbe.uns.ac.rs (J.P.); 2 Department of Biotechnology, University of Szeged, Temesvari krt. 62, H-6726 Szeged, Hungary; zsolt.doffkay@bio.u-szeged.hu (Z.D.); rakhely@brc.hu (G.R.)

**Keywords:** Pseudomonas phage Delta, *Pseudomonas virus PaP3*, genome ends, bacteriophage insensitive mutants (BIM), bacterial virulence factors, antibiotic resistance genes, human allergens, therapeutic phage selection

## Abstract

The applicability and safety of bacteriophage Delta as a potential anti-*Pseudomonas aeruginosa* agent belonging to genus *Bruynoghevirus* (family *Podoviridae*) was characterised. Phage Delta belongs to the species *Pseudomonas virus PaP3*, which has been described as a temperate, with *cos* sites at the end of the genome. The phage Delta possesses a genome of 45,970 bp that encodes tRNA for proline (Pro), aspartic acid (Asp) and asparagine (Asn) and does not encode any known protein involved in lysogeny formation or persistence. Analysis showed that phage Delta has 182 bp direct terminal repeats at the end of genome and lysogeny was confirmed, neither upon infection at low nor at high multiplicity of infection (MOI). The turbid plaques that appear on certain host lawns can result from bacteriophage insensitive mutants that occur with higher frequency (10^−4^). In silico analysis showed that the genome of Delta phage does not encode any known bacterial toxin or virulence factor, determinants of antibiotic resistance and known human allergens. Based on the broad host range and high lytic activity against planktonic and biofilm cells, phage Delta represents a promising candidate for phage therapy.

## 1. Introduction

Bacteriophages are potential therapeutic agents against multidrug and pandrug resistant bacteria which are used throughout the world [1]. One of the major problems of phage application as antibacterial agents in the past was the incomprehension of bacteriophage biology [2]. The first prerequisite that must be fulfilled before consideration of a phage as a therapy candidate is its obligatorily lytic nature and good lytic efficacy. Temperate phages can integrate their genome into bacterial DNA, and by an imprecise excision during induction (i.e., initiation of a lytic cycle), they can excise bacterial DNA and transfer it into a new bacterial host through specialised transduction [3]. Some of these genes can contribute to the virulence or antibiotic resistance of infected bacteria. Both obligatorily lytic and temperate phages can be responsible for generalised transduction, which occurs during phage DNA packaging into procapsids. Sometimes bacterial DNA is packaged into viral particles, and frequency of this phenomenon primarily depends on the DNA packaging mechanism, which is the most prominent in phages that use the head-full mechanism [4]. In this context, careful phage selection should prevent the transfer of genes encoding virulence factors, human/animal allergens or genes responsible for bacterial resistance to antibiotics [5,6,7,8]. Some phages encode proteins involved in their own life cycle, which also act as eukaryotic toxins or allergens; the best-known example is Vibrio phage CTXphi, whose zonula occludens toxin (Zot) is involved in phage extrusion [9]. So far, many phages have been described, and their genomes were sequenced, including phages of *Pseudomonas aeruginosa*. However, even today, beside a lot of information available, crucial characteristics (e.g., phage temperate or obligatorily lytic nature) are not taken into account during selection for in vivo studies and clinical trials.

*Pseudomonas virus PaP3* (*Viruses*; *Duplodnaviria*; *Heunggongvirae*; *Uroviricota*; *Caudoviricetes*; *Caudovirales*; *Podoviridae*; *Bruynoghevirus*) has been described originally by Tan et al. [10], and beside the type strain Pseudomonas phage PaP3, additional strains have been described later. These phages share phenotypic characteristics: morphotype C1, with short tail; broad lytic activity against various strains of *P. aeruginosa* and high in vitro lytic efficacy. It is interesting to point out that a phage belonging to genus the *Bruynoghevirus* is a part of a commercial preparation for human application (e.g., Intesti-bacteriophage, Georgia) [11]. However, some properties important for the phage therapeutic application have not yet been described, including involvement in production of toxins, allergens or antibiotic resistance factors. On the other hand, some published data are controversial; for instance, Pseudomonas virus PaP3 is described as a temperate phage with cohesive ends (*cos*) [10], while *Pseudomonas virus Luz24*, was described as an obligatorily lytic phage with direct terminal repeats (DTR), lacking the *cos* site [12]. 

The study aimed to examine genome of phage Delta, a new strain of species *Pseudomonas virus PaP3*, and other properties important from the aspect of phage therapy, by determining the phage integrative properties, genome ends and potential presence of genes for bacterial toxins, antibiotic resistance and known human allergens. Thus, here we elucidated phage Delta safety for therapeutical application.

## 2. Materials and Methods

### 2.1. Phage Preparation

The previously isolated Pseudomonas phage Delta [13] was multiplied using original host *Pseudomonas aeruginosa* strain PA-4U, precipitated in PEG6000 and NaCl, and purified in CsCl by density ultracentrifugation, as described previously [13,14]. The phage suspension was dialyzed and further treated with DNase and RNase. The purified phage suspension was used for all experiments.

### 2.2. DNA Sequencing

DNA was isolated from virions using standard phenol-chloroform procedure [15]. The isolated DNA was treated with RNase and re-precipitated. Whole genome sequencing of phages was performed using Illumina technology, while de novo assembly of sequenced fragments was carried out by CLC Genomics Workbench 6.5 software and Mira 4.

### 2.3. Phage Delta Genome Analysis

Open reading frames (ORF) in phage Delta genome were predicted using GenemarkS [16] and MyRast [17]. The genomic DNA was compared using BLASTN algorithm with other related strains and species of genus *Bruynoghevirus*, as well as from other related genera (*Krylovvirus*, *Vicosavirus* and *Bjornvirus*). Phages of the same species share >95% DNA similarity (identity × query coverage) and phage exemplars belonging to the same species are considered phage strains [18].

The whole genome sequences of bacteriophages were obtained from GenBank and used for phylogenetic analysis (listed in Table 1). Phage phylogeny was examined using MUSCLE alignment and phylogenetic tree was constructed using MEGAX [19]. The major capsid protein and terminase amino-acid sequences were compared among the phages using ClustalW alignment and Maximum Likelihood method with Kimura 2-parameter model and bootstrap value 1000 [20]. 

The presence of tRNA genes in the genomes were predicted for phage Delta and (re)checked for other related phages using ARAGORN (http://mbio-serv2.mbioekol.lu.se/ARAGORN/, accessed on 6 June 2016) [21] and tRNAScanSE Search Server (http://lowelab.ucsc.edu/tRNAscan-SE/, accessed on 16 May 2016) [22]. BLASTN algorithm was used to determine PaP3 related prophage existence in sequenced genomes of *Pseudomonas* strains available in GenBank. The lifestyle of the phages was predicted using the PHACTS algorithm (http://www.phantome.org/PHACTS/, accessed on 18 July 2016) [23]. 

### 2.4. Lysogeny Formation

To examine possibility of phage Delta integration into bacterial DNA, phages and sensitive *P. aeruginosa* PAO1 strain were incubated for 24 h at 37 °C at MOI 0.1 and 10. The mixture was then plated on a Luria-Bertani medium to obtain colonies of survived bacterial cells. Several colonies were picked up, transferred onto new medium and subsequently their DNA was isolated using GeneJET Genomic DNA Purification Kit (Thermo Fisher Scientific*,* Vilnius, Lithuania). The extracted DNA was digested using FastDigest endonuclease SmaI (Thermo Fisher Scientific, Inc., Waltham, MA, USA), which cannot cut phage Delta DNA but can cut bacterial DNA, generating many small-sized fragments. In parallel, DNA of non-infected bacteria was used as a control of enzyme activity and absence of phage Delta DNA in the bacterial genome, while phage DNA was used as a negative control of enzyme activity (i.e., control of phage DNA integrity after SmaI treatment). DNA fragments were visualised on 0.7% agarose gels with ethidium bromide under UV light and documented (BioDoc Analyse, Biometra, Germany).

### 2.5. Bacteriophage Insensitive Mutants (BIM)

The incidence of phage Delta insensitive mutants of *P. aeruginosa* was determined using a modified method by O’Flynn et al. (2006) [24]. Two bacterial strains were used for the experiment: PA-4U, a urine isolate UB-5296 and ear-infection strain OB-7025 [13]. To obtain MOI = 10, 1 × 10^10^ CFUmL^−1^ of bacteria and 1 × 10^11^ PFUmL^−1^ of bacteriophage were added. Based on the Poisson distribution, MOI of 10 gives 100% infection in a culture, so only BIM can survive. The mixture of phage and bacteria was incubated at 37 °C for 15 min and then various volumes were poured over solid LB medium. The plates were incubated at 37 °C, and the number of survived cells was determined by counting colonies after 24 and 48 h. The mean colony numbers and standard deviations were calculated from three independent experiments with three replicates. The frequency of occurrence of mutants was calculated as the ratio of the number of grown colonies and the initial number of bacteria per milliliter. Phage resistance and absence of (pro)phage genome in cells were additionally performed.

### 2.6. Determination of Phage Genome Ends

The presence of *cos* sites at the ends of phage Delta genomic DNA was examined by the coherence of the two terminal restriction fragments [25]. For this purpose, DNA was restricted by BamHI that gives a few well distinctive bands. The restricted DNA was heated at 75 °C 15 min and then cooled either slowly at room temperature or rapidly on ice. The slow cooling conditions lead to annealing of cohesive ends, visible on an agarose gel as an appearance of a larger size band, with loose or fainting of two smaller sized bands. Under fast cooling conditions, the two terminal bands do not have time to anneal, giving no change of the original RFLP pattern. The products were analysed by 1.0% agarose gel electrophoresis detected with ethidium bromide under UV light and documented (BioDoc Analyse, Biometra, Göttingen, Germany).

### 2.7. Potential Virulence Factors

Potential virulence factors encoded by phages were examined by BLASTP algorithm against a virulence factor database (VFDB, http://www.mgc.ac.cn/VFs/main.htm, accessed on 17 July 2016) [26]. Hits with more than 70% coverage and 30% identity were considered as positive results or if E < 10^−3^.

### 2.8. Antibiotic Resistance Genes

Potential antibiotic resistance genes in the genome of *Pseudomonas virus PaP3* were examined by BLASTP against the resistance gene database, with setup value >10% identity and E < 10^−3^ (https://card.mcmaster.ca/analyze/blast, accessed in 5 July 2016) [27].

### 2.9. Potential Allergens

The phage proteins were analysed to identify potential human allergenic proteins, using tools available at http://www.allergenonline.com (accessed on 15 July 2016) from the Food Allergy Research [28]. For the full-length alignments by BLASTP a possible cross-reactivity was considered if E < 10^−3^. An additional method, using a sliding window of 80 amino acid segments of each protein, was also carried out to confirm significance. The best identities of phage proteins and allergens greater than 35% and E < 10^−3^ were considered significant [29].

## 3. Results and Discussion

Phage Delta morphology corresponds to other strains of the species *Pseudomonas virus PaP3* with the head diameter of approx. 60 nm and morphotype C1 [25]. The genome nucleotide sequence was deposited in the GenBank database under accession number MG432151, and it consists of 45,970 bp with GC% 52.2 (Table 1). Three tRNA were detected: tRNA- proline (Pro), asparagine (Asn) and aspartic acid (Asp), as in other strains of *Pseudomonas virus PaP3*, with an exception of PaP3 phage that encodes tRNA for tyrosine (Tyr) in addition. The genome encodes 69 proteins and approximately one third of genes are in opposite orientation in comparison to the rest of the genome. The genome comprises early genes, genes for DNA synthesis/replication, virion assembly and host lysis but lacks integrase, repressor and known genes involved in prophage persistence. 

**Figure 1:**
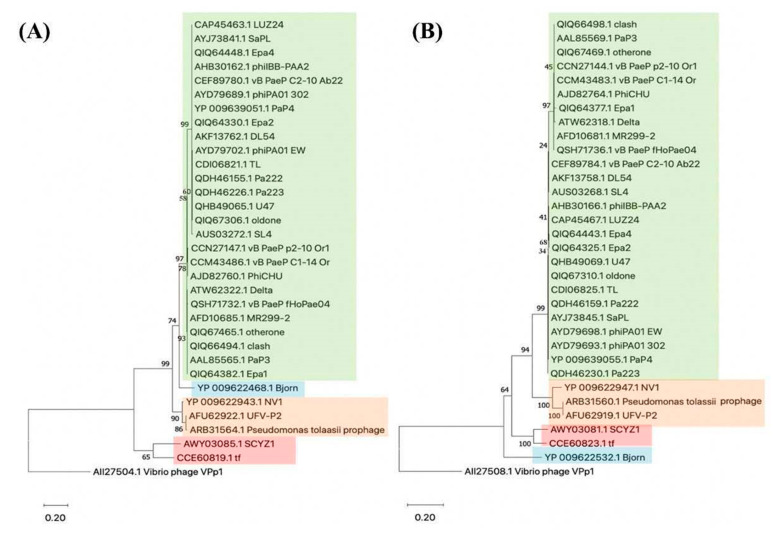
Phylogenetic relationships of phage Delta with other related phages (members of *Bruynoghevirus*—green, *Krylovvirus*—red, *Vicosavirus*—orange and *Bjornvirus*—blue) and a prophage of *P. tolassii*, based on amino-acid sequences of major coat protein (**A**) and terminase (**B**). The evolutionary history based on protein sequences was inferred using the Maximum Likelihood method and JTT matrix-based model. Initial trees for the heuristic search were obtained automatically by applying Neighbor-Joining and BioNJ algorithms to a matrix of pairwise distances estimated using a JTT model, and then selecting the topology with superior log likelihood value. Evolutionary analyses were conducted in MEGA X [18] and the percentage of replicate trees in which the associated taxa clustered together in the bootstrap test (1000 replicates) are shown next to the branches [19]. Vibrio phage VPp1 was used as an outlier.

The phylogenetic analyses based on amino-acid sequences of major head protein (Figure 1A) and terminase (Figure 1B) showed relations of phage Delta to members of the genus *Bruynoghevirus* from the family *Podoviridae*, but also to genera *Krylovvirus*, *Vicosavirus* and *Bjornvirus*. Analyses of DNA similarity of phage Delta sequence confirmed that this phage belongs to the species *Pseudomonas virus Pap3*, and that there are nine different strains in total, along with phage Delta, isolated throughout the world that belong to this species (Table 1).

The bacteriophage Delta has broad lytic activity against many *P. aeruginosa* strains (72.7% from a culture collection) [13] and has prominent lytic activity against both planktonic and biofilm cells [14]. Even though the phage is a promising candidate for phage therapy, its close relative, phage PaP3, was proven to be temperate with genome containing *cos* ends. This characteristic is not desirable from the aspect of phage application in therapy, therefore the phage Delta was further examined.

### 3.1. Is Bacteriophage Delta Temperate or Obligatorily Lytic Phage?

With the exception of PaP3, lysogeny formation was not confirmed neither for other strains of species *Pseudomonas virus PaP3*, nor for other members of the genus *Bruynoghevirus*. 

For PaP3 phage, a sequence in tRNA-Pro was postulated as an *att* site (GGTCGTAGGTTCGAATCCTAC), and it is in accordance with the fact that tRNAs are generally considered an integration site into the bacterial genome [47]. This 21-mer sequence is present in all strains of the species Pseudomonas virus PaP3 and phage phiCHU, while almost all other members of the genus *Bruynoghevirus* have one A→G transition in the sequence (Table 1). Even though this difference can be a reason why lysogeny have not been proven in these phages, enzymes involved in recombination/integration usually are not sensitive to the point mutations [48,49].

The phages of peripherally related genera *Krylovvirus*, *Bjornvirus* and *Vicosavirus* also lack this postulated 21-mer *att* sequence, although *Krylovvirus* encode tRNA in contrast with other two genera. Only for *Vicosavirus* lysogeny formation was confirmed, and it seems that *att* site is different. Namely, when phage Delta genome, or genome of other members of genus *Bruynoghevirus* are compared to *Pseudomonas aeruginosa* group (taxid:136841) or *Pseudomonas* (taxid:287) by BLASTN algorithm *in silico*, no significant similarities were detected, i.e., *P. aeruginosa* available genomes do not contain sequences similar to these phages (the results are not shown). Among the sequenced genomes of various *Pseudomonas* species, only one prophage of *Pseudomonas tolaasii* 2192T showed some similarity to phages of genus *Bruynoghevirus* (31.8–36.2%), and significant similarity with members of genus *Vicosavirus* (72.1–89.3%) (Table 1 and Figure 1). The prophage in *P. tolaasii* genome, related to *Bruynoghevirus*, neither encode tRNAs nor integrates in the proximity of bacterial tRNA. For instance, prophage integration occurs at a tRNA gene for phages carrying Lambda and P4-like integrases, although they do not encode any tRNA, but only small parts of the gene [50]. However, Bailly-Bechet et al. (2007) [51] indicate that tRNA presence in phage genome is not primarily because of integration but found a significant association between tRNA distribution and codon usage, as phage tRNA codons are simultaneously highly used by the phage genes, being rare in the host genome. They even found that obligatorily lytic phages contain more tRNAs than temperate ones. Just as a comparison, the well-known obligatorily lytic T4 also encodes tRNA [52], without ability to form lysogeny. Thus, the presence of tRNA genes in *Bruynoghevirus* not necessarily indicates their temperate nature.

The alignment of postulated *P. tolaasii* 2192T prophage ends indicated 42-mer *att* site with sequence GATGCAGATGGGCGTAATGCTCAACAAGAACCGCGAGGCTGC (Table S1), which is different from postulated *att* site of PaP3 related phages, and can be found only in *Vicosavirus* members (Table 1). Accordingly, even if the phages from the genus *Bruynoghevirus* integrate into the host DNA, the integration site is different than for *Vicosavirus*.

Furthermore, it seems that some phages of genus *Bruynoghevirus* contain genes potentially involved in lysogeny formation. For instance, a protein conserved region from SPFH superfamily (stomatin, prohibitin, flotillin and HflK/C) can be detected in some related viruses, including the prophage of *P. tolaasii* (Table 1). This protein is frequently annotated as „transposase fusion protein“. The similar protein HflK/C (High frequency of lysogenization) plays a role in the decision making between lysogenic and lytic cycle during Lambda phage infection, antagonising activity of FtsH ATPase/protease, that in *E. coli* is involved in degradation of Lambda phage cII transcriptional activator, responsible for lysogenisation [53,54]. The role of this protein is still not clear, since some phages that are characterised as obligatorily lytic encode it, such as Pseudomonas phage KPP10, PAK-P3, P3-CHA, CHA_P1 and PAK_P5 (*Myoviridae*), which were successfully used in a cystic fibrosis mice model [55,56,57]. Thus, phages that encode this protein may be temperate, but its presence in genome is not necessary confirmation of phage temperate nature. Furthermore, the analysis showed that the protein with the conserved SPFH sequence is absent in all strains of *Pseudomonas virus PaP3*, in phages phiCHU, Luz24, phiBB-PAA2, Epa2 (genus *Bruynoghevirus*), tf (genus *Krylovvirus*) and Bjorn (genus *Bjornvirus*) (Table 1).

Even the lack of phage encoded integration enzymes in other PaP3 related phages does not, however, undoubtedly confirm their obligatorily lytic nature, as prophages can be formed by the activity of corresponding host enzymes [58]. For type strain PaP3, it has been previously demonstrated that upon *P. aeruginosa* PAO1 infection, the phage genome can be detected as an uncut band by PstI endonuclease in bacterial genomic DNA.

**Figure 2:**
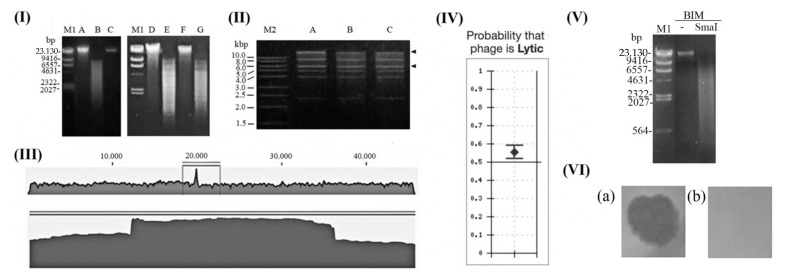
Determination of Pseudomonas phage Delta properties and lifestyle: (**I**) PAO 1 genomic DNA (A, D and F) was treated with SmaI (B) generating numerous fragments of small size; phage Delta DNA was not cut by SmaI (C); after PAO1 infection at MOI = 0.1 phage Delta genome was not detected in PAO1 genome (E), and similar was confirmed when PAO1 was infected at MOI = 10; (**II**) phage Delta DNA cut by BamHI show no change of restriction pattern after fast and slow cooling, indicating absence of *cos* site; (**III**) DNA sequence of phage Delta showed elevation of reads typical for direct terminal repeats; (**IV**) PHACTS algorithm showed that phage Delta is obligatorily lytic, with high confidence; (**V**) uncut and SmaI-cut BIM genome showed absence of phage DNA and thus lysogeny; (**VI**) lysis of sensitive *P. aeruginosa* lawn by phage Delta (**a**) and absence of lysis of BIM (**b**).

Using the SmaI enzyme, which cannot cut phage Delta genome, we have not confirmed phage genome in bacterial DNA by RFLP upon infection of PAO1 strain (Figure 2II). The absence of the phage DNA band confirmed a lack of its integration or extrachromosomal presence in PAO1, both after infection with low (MOI = 0.1) or high number of virions (MOI = 10). The two different MOIs were used, as it was confirmed that phage lambda, a model for temperate phages, increases the frequency of lysogeny at higher MOI [59]. A study of global transcriptomic analysis of *P. aeruginosa* PAO1 after infection with PaP3 at MOI = 10 indicate lytic cycle, according to the gene expression and the one-step growth curve, that reach a plateau after 80 min. with burst size approx. 30 [60], resembling to a lytic cycle. Alemayehu et al. (2015) examining phage strain MR299-1 indicated that a previous study calls into question the temperate nature of PaP3 [12], since no site-specific recombinase is encoded up- or downstream of the *attP* site, and immunity or reactivation of the prophage was not demonstrated.

The application of PHACTS showed that all strains of species *Pseudomonas virus PaP3* are obligatorily lytic, and the findings are confident for strains Delta (Figure 2V) and MR299-2, but also for some other species of the genus *Bruynoghevirus* (Table 1). Similar pertains to *Krylovvirus* and *Bjornvirus*, but *Vicosavirus* members are characterised as temperate (phage UVF-P2 with high confidence). This additionally contributes to the obligatorily lytic nature of *Bruynoghevirus* members, including phage Delta.

The turbid plaques produced by members of *Bruynoghevirus* on lawn of certain strains cannot be simply explained by lysogeny formation. To elucidate this phenomenon, the frequency of BIM was determined (Table 2). For original host PA-4U, the frequency of mutant appearance was of order of magnitude 10^−7^, and for heterologous strains UB-6259 and OB-7025 frequencies were 10^−6^ and 10^−4^, respectively. The frequency of BIM was not significantly changed after prolonged incubation. Since phage Delta form turbid plaques on OB-7025 lawn, high frequency of BIM can partially provide an explanation for incomplete lysis and faint plaques formation. The results indicate necessity to examine all host-phage system to determine BIM frequency and to apply measures to decrease BIM frequency (by using phage cocktails, combining phages and antibiotics etc).

Taking into account the above considerations, it is clear that there is no evidence that strains of the species Pseudomonas virus Pap3 are temperate. Even if these phages are temperate, their lysogeny is extremely unstable or integration is very rare.

### 3.2. Is Pseudomonas Phage PaP3 Prone to Generalised Transduction?

There are several types of phage termini: cohesive ends (5′- or 3′-single-strand extensions), circularly permuted direct terminal repeats, short or long exact direct terminal repeats (DTR), terminal host DNA sequences or covalently bound terminal proteins. A particular termini type is a result of a phage packaging strategy: 5′*cos* (e.g., Lambda phage), 3′*cos* (e.g., phage HK97), headful with a *pac* site (e.g., phage P1), headful without a *pac* site (e.g., phage T4), DTR (e.g., phage T7) and host fragment at genome ends (e.g., phage Mu) [25,61]. The bacteriophages that pack DNA by means of headful mechanism can more frequently form defective virions, carrying parts of bacterial DNA, being prone to generalised transduction [61,62]. This is not a desirable property for a phage intended for therapy, since phages can transfer genes involved in bacterial virulence. Experiments with phage Delta genomic DNA restriction pattern with BamHI showed no difference if samples heated to 75 °C and then cooled quickly or slowly (Figure 2III), indicating the absence of *cos* site. A 20-mer sequence postulated as a *cos* in PaP3 (5′-GCCGGCCCCTTTCCGCGTTA-3′) [10] is present in most phage genomes that encode tRNA-Pro (Table 1), identical or with one base changed. The analysis of the phage Delta genome sequence did not confirm *cos* genome ends. Still, it revealed presence of 182 bp long DTR in the genome assembly (Figure 2IV), which is obviously as twice as the average sequence coverage [62].

It is believed that mechanism of packaging and type of termini can be successfully predicted from amino-acid sequence of large terminase subunits, which is quite conserved among the most tailed-phage proteins [63]. Previous analyses of phage LUZ24 and PaP3 terminases are strongly related to the terminase of phage P22, which catalyses the headful packaging, leading to both terminal redundancy and circular permutations [12,64]. However, some studies showed that terminase amino-acid sequence is not very reliable in determination of packaging mechanism [65], which is confirmed for Luz24, as well as phage Delta, that both have long direct terminal repeats (182 and 184 bp, respectively). Summarising our results on genome ends examination, it is clear that phage Delta has a packaging mechanism similar to phage T7, being less prone to generalised transduction than phages with a headful packaging strategy. Finally, even if some phage Delta related phages have *cos*, they also rarely mediate in horizontal transfer by generalised transduction as phages with DTRs [66].

### 3.3. Does the Phage Delta Genome Encode Undesirable Proteins?

The results of phage protein in silico examination, carried out to find genes for potential virulence factors, antibiotic resistance proteins and allergens, are presented in Supplementary Materials.

Only one protein encoded by members of *Pseudomonas virus PaP3* showed some similarity with a bacterial virulence factors—Dot/Icm type IV secretion system effector SidK of *Legionella pneumophila* subsp. *pneumophila*, but below cut-off E value. In the phages, this protein is a small terminase subunit and has no conserved domains similar to the virulence factor. Thus, the phages do not encode known bacterial virulence factors.

Analysis of known antibiotic resistance genes showed that almost all members of genus *Bruynoghevirus* possess a gene similar to B3 class of beta lactamase, i.e., metallo-beta lactamase found in *Massilia oculi*. Similarly, members of *Vicosavirus* also encode a protein similar to class A beta lactamase precursor RCP found in *Rhodopseudomonas capsulata*. However, the observed similarities are not significant, indicating that phages do not carry known antibiotic resistance genes.

It is well known that phages do not cause allergic reactions after application, but since their structural proteins are antigenic [67,68], the potential production of IgE should not be neglected. The full-length BLASTP alignments and 80-mer sliding method showed similarity of PaP3, phiCHU and UFV-P2 prroteins to 30 kDa salivary gland allergen variant 2 of *Aedes aegypti*. In addition, ORF 54 of phage PaP4 is significantly similar to collagen alpha-2(I) chain isoform X1 of *Salmo salar*, and ORF 53 of phage tf is significantly similar to high molecular weight glutenin subunit 10 of *Triticum aestivum*. According to the FAO/WHO 2001 experts and the Codex Alimentarius Commission (2003), a >35% identity to an allergen over of any segment of 80 or more amino acids indicates possible cross-reactivity. Even though phages are generally safe for application [69,70], if these phages are intended for therapy, further evaluation of IgE reactivity or clinical cross-reactivity may be warranted to test for these potential allergens. In potential clinical trials, all these phages should be used with precautions in individuals allergic to corresponding antigens.

The results indicate that phage Delta proteins show no significant similarity to known bacterial virulence factors, determinants of *P. aeruginosa* resistance to conventional antibiotics and allergens.

## 4. Conclusions

For most *Bruynoghevirus* members, including phage Delta, there is no evidence of lysogeny formation, and DNA is packaged by the T7 model, indicating lower generalised transduction capacity. The genome of the Delta phage does not encode known virulence factors, antibiotic resistance determinants and allergens. These data, along with high lytic efficacy and broad activity against various strains of *P. aeruginosa* recommend this phage as a potent anti-*Pseudomonas* agent. Some shortfalls, such us high frequency of BIM can be overcome by phage cocktail preparation, combination with antibiotics etc. If such thorough analysis of a phage does not confirm undesirable properties, for the therapeutic phages should be applied „presumption of innocence—everyone is considered innocent until proven guilty“, i.e., a phage that fulfils criteria for application is considered safe for therapy until proven otherwise.

## Figures and Tables

**Table 1:** Characteristics of Pseudomonas phage Delta and related (pro)phages.

Genus in Family *Podoviridae*	Species	Strain ^1^ Access. No.	Genome (bp)/No. CDS	Origin (Country)	Plaques (mm)	Head Diameter (nm)	tRNA	Similarity to Delta (%)	HflK/C/cos ^1^	Integrat. Tolaasii/PaP3	Phage Lifestyle ^2^	Ref.
*Bruynoghevirus*	*Pseudomonas virus PaP3*	**PaP3** AY078382	45,503/72	Hospital sewage/China	Turbid (1.5)	55	Asn, Asp, Pro, Tyr	96.2	N/Y	N/Y	0.51 ± 0.04 L	[10]
		C1-14_Or HE983844	45,469/64	Sewage water/France	N. A. ^3^	60	Asn, Asp, Pro	96.3	N/Y	N/Y	0.51 ± 0.03 L	[30]
		P2-10_Or1 HF543949	44,030/71	Eliava “Pyophage”/Georgia	N. A.	58–60	Asn, Asp, Pro	96.8	N/Y	N/Y	0.52 ± 0.05 L	[30]
		MR299-2 JN254801	44,789/68	Sewage from water treatment plant	N. A.	40–60	Asn, Asp, Pro	96.6	N/Y	N/Y	0.53 ± 0.03 L *	[31]
		otherone MT119373.1	44,930/67	Wastewater	N.A.	N.A.	Asn, Asp, Pro	96.9	N/Y	N/Y	0.53 ± 0.04 L	[32]
		Clash MT119362.1	44,912/67	Wastewater	N.A.	N.A.	Asn, Asp, Pro	96.8	N/Y	N/Y	0.53 ± 0.06 L	[32]
		Delta MG432151.1	45,970/69	Municipal wastewater/Serbia	Clear/turbid (2.5–5.0)	63	Asn, Asp, Pro	100.0	N/Y	N/Y	0.54 ± 0.03 L *	This ref.
		vB_PaeP_fHoPae04 MW329986.1	45,491/70	Hospital wastewater, Finland	N.A.	N.A.	Asn, Asp, Pro	96.4	N/Y	N/Y	0.53 ± 0.03 L	[33]
		Epa 1 MT108723.1	45,230/67	N.A.	N.A.	N.A.	Asn, Asp, Pro	94.3	N/Y	N/Y	0.52 ± 0.04 L	N.A.
	*Pseudomonas virus CHU*	**CHU** KP233880.1	45,626/76	Pond/Russia	Variable	N.A.	Pro, Asp, Asn	92.1	N/Y	N/Y	0.54 ± 0.06 L	[34]
	*Pseudomonas virus Pa223*	**Pa223** MK837012.1	45,703/71	N.A.	N.A.	N.A.	Asn *, Tyr *, Pro *	78.4	Y/Y	N/Y (1) *	0.58 ± 0.05 L *	[35]
	*Pseudomonas virus Luz24*	**Luz24** AM910650.1	45,503/68	Hospital sewage/Belgium	Clear/turbid (1.0–5.0)	63	Asn *, Tyr *, Pro *	74.3	N/N	N/Y (1)	0.66 ± 0.12 L *	[12]
	*Pseudomonas virus Dl54*	**DL54** KR054029.1	45,673/71	crude sewage or flood water/UK	Clear/turbid	45	Ile *, Asp *, Pro *	77.1	Y/Y	N/Y (1)	0.53 ± 0.07 L	[36]
	*Pseudomonas virus C2-10_Ab22*	**C2-10_Ab22** LN610578.1	45,808/71	Carrefour de l’Indénié/Ivory Coast	N.A.	N.A.	Pro, Tyr, Asn	74.8	Y/N	N/Y (1)	0.55 ± 0.08 L	[30]
	*Pseudomonas virus phiBB-PAA2*	**phiBB-PAA2** KF856712.1	45,344/66	Hospital sewage/Portugal	N.A.	N.A.	Pro, Asp, Ile	81.8	N/Y	N/Y (1)	0.58 ± 0.06 L *	[37]
	*Pseudomonas virus Pap4*	**Pap4** KC294142.1	43,895 */70	N.A.	Transparent (3.0–5.0)	50	No	75.3	Y/Y	N/N	0.58 ± 0.05 L *	[38]
		phiPAO1-EW MG589386.1	46,403/71	N.A.	N.A.	N.A.	Ile, asp, Pro	75.5	Y/Y	N/Y (3)	0.56 ± 0.05 L *	N.A.
		phiPAO1_302 MG589385.1	46,093/70	N.A.	N.A.	N.A.	Pro, Tyr, Asp, Ile	65.3	Y/Y	N/Y (1)	0.54 ± 0.09 L	N.A.
		SaPL MH973725.1	45,796/63	Samanabad Canal, Lahore/Pakistan	Transparent (4.0–5.0)	N.A.	Asn *, Asp *, Tyr *, Pro *	64.5	Y/Y	N/Y (1)	0.56 ± 0.07 L	[39]
		EPa4 MT118288.1	45,439/53	N.A:	N.A.	N.A.	Asn *, Tyr *, Pro *	64.9	Y/Y	N/Y (1)	0.56 ± 0.05 L *	[40]
		Pa222 MK837011.1	45,770/58	N.A.	N.A.	N.A.	Asn *, Asp *, Tyr *, Pro *	78.4	Y/Y	N/Y (1)	0.56 ± 0.06 L *	[35]
		Oldone MT119371.1	45,313/70	Wastewater	N.A.	N.A.	Asn *, Asp *, Tyr *, Pro *	78.8	Y/Y	N/Y (1)	0.52 ± 0.07 L	[32]
		U47 MN562749.1	43,444/68	N.A.	N.A.	N.A.	Asn *, Asp *, Tyr *, Pro*	78.2	Y/Y	N/Y (1)	0.59 ± 0.07 L *	N.A.
		Epa 2 MT108724.1	43,229/51	N.A.	N.A.	N.A.	Asn *, Tyr *, Pro *	78.0	N/Y	N/Y (1)	0.53 ± 0.06 L	[41]
		TL HG518155.1	45,696/65	N.A.	Transparent turbid, large		Asn *, Tyr *, Pro *	73.3	Y/N	N/Y (1)	0.55 ± 0.08 L	[42]
		SL4 MF768469.1	44,194/65	Hospital sewage, Germany	N.A.	55	Asn *, Tyr *, Pro *	76.4	Y/N	N/Y (1)	0.54 ± 0.05 L	[43]
*Krylovvirus*	*Pseudomonas virus tf*	**tf** HE611333	46,271/72	N.A.	N.A.	N.A.	Tyr *	23.1	N/N	N/N	0.52 ± 0.04 L	[44]
	*Pseudomonas virus SCZY1*	**SCYZ1** MH518298.3	47,475/62	Soil	N.A.	N.A.	Pro *	2.3	Y/N	N/N	0.51 ± 0.04 L	N.A.
*Vicosavirus*	*Pseudomonas virus NV1*	**NV1** NC_042107.1	45,058/64	Untreated sewage, River Thames, UK	Hazy(<2)		No	35.0	Y/N	Y (1)/N	0.52 ± 0.05 T	[45]
	*Pseudomonas virus UVF-P2*	**UVF-P2** JX863101	45,517/75	Dairy industry wastewater, Brazil	N.A.	N.A.	No	34.7	Y/N	Y (2)/N	0.53 ± 0.03 T *	[46]
		Prophage CP020369.1 (6815074.. 6769117)	52.973/85	-	-	-	No		Y/N	Y/N	0.50 ± 0.04 T	This ref.
*Bjornvirus*	*Pseudomonas virus Bjorn*	**Bjorn** NC_042103.1	45,936/69	Plant compost	N.A.	N.A.	No	20.0	N/N	N/N	0.53 ± 0.05 L	N.A.

^1^ Type strain is written in bold; ^2^ Y—yes; N—no; number in parenthesis represent number of different nucleotides; ^3^ L—obligatorily lytic phages; T—temperate phages; Asterix (*) represent high confidence; N.A.—not available.

**Table 2:** Frequencies of phage Delta insensitive mutant appearance of various strains.

	BIM Frequency	
Bacterial Host	after 24 h	after 48 h
PA-4U	3.30 × 10^−7^ ± 3.12 × 10^−7^	5.38 × 10^−7^ ± 5.56 × 10^−8^
UB-2596	2.36 × 10^−6^ ± 2.74 × 10^−7^	5.67 × 10^−6^ ± 1.67 × 10^−6^
OB-7025	7.95 × 10^−4^ ± 3.24 × 10^−5^	9.62 × 10^−4^ ± 4.12 × 10^−5^

## Data Availability

Not applicable.

## Supplementary Materials

The following are available online at https://www.mdpi.com/article/10.3390/v13101965/s1, Table S1: Predicted PaP3 related prophage in genome of *P. tolaasii* 2192T using various tools for prophage detection and manually predicted, Table S2: Potential virulence factors, antibiotic resistance related proteins and allergens encoded by phage Delta and other related (pro)phages.
