# Supplementary material for: Phages from Genus Bruynoghevirus and Phage Therapy: Pseudomonas Phage Delta Case
Source: Viruses. 2021 Sep 30;13(10):1965. doi: 10.3390/v13101965 (PMC8540360; doi:10.3390/v13101965)
Supplement: Supplementary file 1 [file viruses-13-01965-s001.zip › Table S1.pdf]

Table S1. Predicted PaP3 related prophage in genome of *P. tolaasii* 2192T using various tools for prophage detection and manually predicted

Prophage in *P. tolaasii* (https://edwards.sdsu.edu/PhiSpy/upload.php?)				
Tool	Coordinates	Size (kbp)/proteins	Score	Completeness
PHASTER[1]	6801317..6801790	54.6/76	140	intact
Prophage Hunter[2]	6769076..6815378	46.3/67	0.87/1	active
	6815378..6810263	24.5/39	0.73	ambiguous
Profinder[3]	6763248 - 6815387	52.1/73	41.2	N.A.
Manually predicted[4]	6762867.. 6815803	52.9/72	-	-

[1] Arndt, D., Grant, J., Marcu, A., Sajed, T., Pon, A., Liang, Y., Wishart, D.S. (2016) PHASTER: a better, faster version of the PHAST phage search tool. Nucleic Acids Res., 2016 May 3. https://phaster.ca/

[2] Song, W., H. X. Sun, C. Zhang, L. Cheng, Y. Peng, Z. Deng, D. Wang, Y. Wang, M. Hu, W. Liu, H. Yang, Y. Shen, J. Li, L. You and M. Xiao (2019). "Prophage Hunter: an integrative hunting tool for active prophages." Nucleic Acids Res. https://doi.org/10.1093/nar/gkz380 https://pro-hunter.genomics.cn/

[3] Lima-Mendez, G., van Helden, J., Toussaint, A., Leplae, R. 2008. Prophinder: a computational tool for prophage prediction in pro-karyotic genomes Bioinformatics http://aclame.ulb.ac.be/Tools/Prophinder/aclame_hits.html

[4] Manually prediction was based on the alignment (a part is shown below)

```
gb|CP020369.1|:6813515-6816088      ACTTGACCGCCAGATGCAGATGGGCGTAATGCTCAACAAGAACCGCGAGGCTGCCTTGGA      1607
gb|CP020369.1|:6766573-6781964      CAGCTCCCACACGATGCAGATGGGCGTAATGCTCAACAAGAACCGCGAGGCTGCTCTGGA      2551
                                          ** *  ******************************************  ****

gb|CP020369.1|:6813515-6816088      TCGTCAACTTAAGGAGCGTGAGGTTACTGTCAAGGAGAAGACTGGTGACTCCCAAGTCAA      1667
gb|CP020369.1|:6766573-6781964      TCGTCAGCTCAAGGAACGTGAGGTTACAGTCAAGGAGAAGACTGGGGATTCCCAAGTGAA      2611
                                    ****** ** ***** *********** ***************** ** ******** **

gb|CP020369.1|:6813515-6816088      AGTAGGTGAGGGTAACCTTGCTGTCAACCAAGGTAAACTTGCGAATGACACTACCAAGAC      1727
gb|CP020369.1|:6766573-6781964      GGTAGGTGAGGGCAACCTCGCTGTCAACCAAGGGAAGCTTAAGAACGACACCGTTAAAAC      2671
                                     *********** ***** ************** ** ***  *** *****    ** **

gb|CP020369.1|:6813515-6816088      CAAGGGCATCATCACCAAGTGGGACAACGACAACCGTAATGCTCAGGCATCTAATGCCAC      1787
gb|CP020369.1|:6766573-6781964      TGGTGGCATCCTGAACAAGTGGGACAACGATAACAAGAACGCTCAAGCTGCTAACGCTAC      2731
                                        ****** * * *************** ***   ** ***** **  **** ** **

gb|CP020369.1|:6813515-6816088      CAAGCGCTATGGTATTGACGTGGGTGCATCCACAGCTATCCGTGGTCAAGACCTCCGGGC      1847
gb|CP020369.1|:6766573-6781964      CAAGCGCTACGGTATTGACGTTGGGGCATCTACAGCTATCCGTGGTCAAGACTTGCGTGA      2791
                                    ********* *********** ** ***** ********************* * ** *

gb|CP020369.1|:6813515-6816088      TCAGACTGCTGCTGCTGGTCGCTCTANNNNNNNNNNNNNNNNNNNNNNNNNNNNNNNNNN      1907
gb|CP020369.1|:6766573-6781964      TCAAGCTGCTGCTGCTGCTCGTGGTACCACTGAACGTGGTCAAGACATGACTCAAGAGAC      2851
                                    ***  ************ ***   **

gb|CP020369.1|:6813515-6816088      NNNNNNNNNNNNNNNNNNNNNNNNNNNNNNNNNNNNNNNNNTGCTCGTCAGAACAACGAA      1967
gb|CP020369.1|:6766573-6781964      TG--------CCAAGCTTCGTACCGCGGCTGACTTGTCTAAAGCACGTCAACGTAACGAG      2903
                                                                              ** *****    *****

gb|CP020369.1|:6813515-6816088      ACCAAACTGGCTGCTGCTCAACTCCGTGCTAAGGCTTCCGAGAAGGCTGCCAAAGGACAA      2027
gb|CP020369.1|:6766573-6781964      ACTACTCTAGCTGCTGCTCAGGTTCGTGCTAAGGCAGCCGAGAAAGCAGCCAAAGGAGAA      2963
                                    ** *  ** ***********  * ***********  ******* ** ********* **

gb|CP020369.1|:6813515-6816088      AGCTTGACAAACAAGGATTTTTCTGGTATAATTGATTCATTCGATGGATCTGATGCTGTT      2087
gb|CP020369.1|:6766573-6781964      AGCTTGACATTTAAGGATTCTTCTGGTATAATTGATAGTATGGATGGGACTGACGCAGTT      3023
                                    *********   ******* ****************    * *****  **** ** ***

gb|CP020369.1|:6813515-6816088      GGTGGTAAGAAGCTTTCTAAAGAAGCTAAGGCCGCCGTGAGCATCTCTGCCCGTAACCGA      2147
gb|CP020369.1|:6766573-6781964      GGTGGCAAGAAGCTTTCGAAAGAAGCTCAAGCTATCGTGAACGTTCAATTCCGTAACCGC      3083
                                    ***** *********** ********* * **   ***** * *      *********

gb|CP020369.1|:6813515-6816088      GTGATCGCCAACCCAGATCAAGATCCCAGAGTGACGGTAGTGGTGTCGATCGAGTCGGTA      2207
gb|CP020369.1|:6766573-6781964      ATGAAGGCAGACCCTACCTCAGACCCAGCAGGTGTTATTGCTGAGCTGTACCAAGGGATC      3143
                                     ***  **  ****      *** **   **      * *  * *  *  * *   * *
```
