# Supplementary material for: Phages from Genus Bruynoghevirus and Phage Therapy: Pseudomonas Phage Delta Case
Source: Viruses. 2021 Sep 30;13(10):1965. doi: 10.3390/v13101965 (PMC8540360; doi:10.3390/v13101965)
Supplement: Supplementary file 1 [file viruses-13-01965-s001.zip › Table S2.pdf]

Table S2. Potential virulence factors, antibiotic resistance related proteins and allergens encoded by phage Delta and other related (pro)phages. The most similar protein is shown in the table, and significantly similar proteins are shown in bold.

Genus	Pseudomonas phage	Toxins	Antibiotic resistance	Alergens	
				Fasta	80-mer sliding
Bruynoghevirus	**PaP3**<br>AY078382	ORF1: VFG045504<br>(sidK) Dot/Icm type IV secretion system effector SidK, interacting with VatA, a key component of the proton pump. Suc [Dot/Icm] [*Legionella pneumophila subsp. pneumophila* str. Philadelphia 1]<br>identity 33%; E=0.042	ORF 16: AJP77057.1<br>MSI-1 is a subclass B3 metallo-beta lactamase isolated from *Massilia oculi* conferring resistance to carbapenems<br>identity 36%, E=0.004	**ORF6: AAB58417.1**<br>**30 kDa salivary gland allergen Aed a 3 [*Aedes aegypti*]**<br>**identity 30.3%; similarity 55.2%, E=0.0008**	**ORF 6: AAB58417.1**<br>**best identity 36.4%, E=0.0008**
	C1-14_Or<br>HE983844	ORF1: same	ORF15: same as above	ORF5: Same as above<br>identity 29.9%; similarity 55.6%, E=0.003	-
	P2-10_Or1<br>HF543949	Same	ORF15; same as above	ORF5: Same as above<br>identity 31.5%; similarity 54.5%, E=0.002	-
	MR299-2<br>JN254801	Same	ORF16; same as above;<br>identity 36%, E=0.003	ORF6: Same as above<br>identity 30.3%; similarity 55.2%, E=0.0015	-
	otherone<br>MT119373.1	-	ORF53; same as above	ORF63: Same as above<br>identity 30.2%; similarity 55.4% E=0.002	-
	Clash<br>MT119362.1	-	ORF53: same as above	ORF63: same as above<br>identity 30.2%; similarity 55.4%, E=0.002	-
	Delta<br>MG432151.1	ORF1: VFG045504<br>(sidK) Dot/Icm type IV secretion system effector SidK, interacting with VatA, a key component of the proton pump. Suc [Dot/Icm] [*Legionella pneumophila subsp. pneumophila* str. Philadelphia 1]<br>identity 33%; E=0.042	ORF 16: same as above	ORF16: CBJ24286.1<br>ragweed homologue of Art v 1 precursor [*Ambrosia artemisiifolia*]<br>identity 38.2%; similarity 61.8%, E=0.0036	-
	vB_PaeP_fHoPae04<br>MW329986.1	-	ORF54; same as above	ORF65: AAB58417.1<br>30 kDa salivary gland allergen Aed a 3 [*Aedes aegypti*]<br>identity 29.1%; similarity 52.7%, E=0.0017	-
	Epa 1<br>MT108723.1	-	ORF16; same as above	ORF16: CBJ24286.1	-

			ragweed homologue of Art v 1 precursor<br>[*Ambrosia artemisiifolia*]<br>identity 39.3%; similarity 64.3%,<br>E=0.0042	
**CHU**<br>KP233880.1	-	ORF 16: same as as above;<br>36%, E=0.004	**ORF 68: AAB58417.1**<br>**30 kDa salivary gland allergen Aed a 3**<br>**[*Aedes aegypti*]**<br>**identity 30.9%; similarity 55.8%,**<br>**E=0.000222**	**ORF 68: AAB58417.1**<br>**best identity 37.80%, E=0.00022**
**Pa223**<br>MK837012.1	-	ORF56; same as above<br>identity 36%, E=0.005	ORF56: CBJ24286.1<br>ragweed homologue of Art v 1 precursor<br>[*Ambrosia artemisiifolia*]<br>identity 40.0%; similarity 60.0%,<br>E=0.0015	-
**Luz24**<br>AM910650.1	-	ORF53; same as above	ORF56: same as above<br>identity 40.0%; similarity 61.8%,<br>E=0.0014	-
**DL54**<br>KR054029.1	-	-	ORF16: same as above<br>identity 40.0%; similarity 61.8%<br>E=0.0025	-
**C2-10_Ab22**<br>LN610578.1	-	ORF 56: same as above<br>identity 36%, E=0.003	**ORF56: same as above**<br>**identity 40.0%; similarity 61.8%**<br>**E=0.00033**	-
**phiBB-PAA2**<br>KF856712.1	-	-	ORF16: ragweed homologue of Art v 1<br>precursor [*Ambrosia artemisiifolia*];<br>CBJ24286.1; id 40.0%; similarity 61.8%<br>E=0.0024	-
**Pap4**<br>KC294142.1	-	-	**ORF 54: XP_013998297.1**<br>**collagen alpha-2(I) chain isoform X1**<br>**[*Salmo salar*]**<br>**identity 30.0%; similarity 54.4%,**<br>**E=0.00038**	**ORF 54: c XP_013998297.1**<br>**best identity 42.0%, E=0.00038**
phiPAO1-EW<br>MG589386.1	-	ORF 19: same as above<br>identity 36%, E=0.005	ORF 18: same as above<br>identity 30.0%; similarity 54.4%<br>,E=0.00042	-
phiPAO1_302<br>MG589385.1	-	ORF55; same as above	ORF 53: XP_013998297.1<br>collagen alpha-2(I) chain isoform X1<br>[*Salmo salar*]<br>identity 29.6%; similarity 53.8%,<br>E=0.0013	-

				ORF55: CBJ24286.1<br>ragweed homologue of Art v 1 precursor<br>[*Ambrosia artemisiifolia*]<br>identity 40.0%; similarity 61.8%,<br>E=0.0013	
	SaPL<br>MH973725.1	-	ORF44; same as above<br>identity 36%, E=0.003	ORF43: CBJ24286.1<br>ragweed homologue of Art v 1 precursor<br>[*Ambrosia artemisiifolia*]<br>identity 40.0%; similarity 61.8%,<br>E=0.0018	-
	Epa4<br>MT118288.1	-	-	ORF 14: AAF75225.1<br>paramyosin isoform, partial [*Anisakis simplex*]<br>identity 28.1%; similarity 54.0%,<br>E=0.0018	-
	Pa222<br>MK837011.1	-	ORF53; same as above;<br>identity 36%, E=0.005	**ORF53: CBJ24286.1**<br>**ragweed homologue of Art v 1 precursor [*Ambrosia artemisiifolia*]**<br>**identity 40.0%; similarity 61.8%,**<br>**E=0.00098**	-
	Oldone<br>MT119371.1	-	ORF55; same as above	ORF55: same as above<br>identity 40.7%; similarity 61.1%<br>E=0.0082	-
	U47<br>MN562749.1	-	ORF52; same as above	ORF52: ragweed homologue of Art v 1 precursor [*Ambrosia artemisiifolia*];<br>CBJ24286.1; id 40.0%; similarity 61.1%<br>E=0.0013	-
	Epa 2<br>MT108724.1	-	-	ORF 48: paramyosin isoform, partial [*Anisakis simplex*] AAF75225.1; id 28.1%;<br>similarity 54.0% E=0.0019	-
	TL<br>HG518155.1	-	ORF51; same as above<br>identity 36%, E=0.005	ORF52: same as above<br>identity 38.2%; similarity 61.8%,<br>E=0.0053	-
	SL4<br>MF768469.1	-	-	ORF36; AAG31026.1<br>subtilisin precursor, partial [*Bacillus licheniformis*]<br>identity 33.1%; similarity 56.1%<br>E=0.0015	-
Krylovvirus	**tf**<br>HE611333	-	-	**ORF 53: CAA31396.1**	**ORF 53: CAA31396.1**<br>**best identity 35.0,% E=0.00054**

				**high molecular weight glutenin subunit 10 [*Triticum aestivum*]**<br>**identity 32.0%; similarity 619%, E=0.00054**	
	**SCYZ1**<br>MH518298.3	-	-	**ORF42: CBJ24286.1**<br>**ragweed homologue of Art v 1 precursor [*Ambrosia artemisiifolia*]**<br>**identity 42.4%; similarity 57.6%, E=0.000067**	-
Vicosavirus	**NV1**<br>NC_042107.1	-	ORF 11: CAA33795.1<br>Class A beta lactamase precursor RCP found in *Rhodopseudomonas capsulata*<br>identity 35%, E=0.002	ORF46: CAA26383.1<br>Gliadin-like protein product [Triticum aestivum]<br>identity 26.1%; similarity 48.6%<br>E=0.0044	-
	**UVF-P2**<br>JX863101	-	ORF 15: Same as above<br>identity 35%, E=0.003	**ORF 61: AAZ23584.1**<br>**HMW glutenin x-type subunit Bx7 [*Triticum aestivum*]**<br>**identity 23.2%; similarity 49.0%, E=0.0003**<br>**ORF70: ABF18122.1**<br>**30 kDa salivary gland allergen variant 2 [*Aedes aegypti*]**<br>**identity 27.9%; similarity 57.5%, E=0.000053**	**ORF70: ABF18122.1**<br>**best identity 35.0%, E=0.00053**
	Prophage<br>CP020369.1<br>(6762867.. 6815803)	ORF1: VFG040073<br>TrbL/VirB6 plasmid conjugative transfer protein [Rvh T4SS] [*Rickettsia australis* Cutlack]<br>identity 39%, E=0.93	ORF 11: Same as above<br>identity 35%, E=0.002	**ORF72: ABF18122.1**<br>**30 kDa salivary gland allergen variant 2 [*Aedes aegypti*]**<br>**identity 25.4%; similarity 52.6%, E=0.000015**	**ORF72: ABF18122.1**<br>**best identity 35.0,% E=0.000013**
Bjornvirus	**Bjorn**<br>NC_042103.1	-	-	**ORF 54: BAN29068.1**<br>**high molecular weight glutenin subunit, partial [*Triticum aestivum*]**<br>**identity 25.8%; similarity 51.6% E=0.00027**<br>**ORF 56: CAA43361.1**<br>**HMW glutenin subunit 1By9 [*Triticum aestivum*]**<br>**identity 27.0%; similarity 50.2%, E=0.000033**	-
